# C9 immunostaining as a tissue biomarker for periprosthetic joint infection diagnosis

**DOI:** 10.3389/fimmu.2023.1112188

**Published:** 2023-02-21

**Authors:** Ann-Kathrin Meinshausen, Jacqueline Färber, Sebastian Illiger, Paolo Macor, Christoph H. Lohmann, Jessica Bertrand

**Affiliations:** ^1^ Department of Orthopaedic Surgery, Otto-von-Guericke University Magdeburg, Magdeburg, Germany; ^2^ Institute of Medical Microbiology, Infection Control and Prevention, Otto-von-Guericke University Magdeburg, Magdeburg, Germany; ^3^ Department of Life Sciences, University of Trieste, Trieste, Italy; ^4^ Health Campus Immunology, Infectiology and Inflammation, Otto-von-Guericke University Magdeburg, Magdeburg, Germany

**Keywords:** periprosthetic joint infection, diagnostic biomarker, C9, inflammatory joint disease, complement pathway

## Abstract

**Background:**

Culture-negative periprosthetic joint infections (PJI) are often false diagnosed as aseptic implant failure leading to unnecessary revision surgeries due to repeated infections. A marker to increase the security of e PJI diagnosis is therefore of great importance. The aim of this study was to test C9 immunostaining of periprosthetic tissue as a novel tissue-biomarker for a more reliable identification of PJI, as well as potential cross-reactivity.

**Method:**

We included 98 patients in this study undergoing septic or aseptic revision surgeries. Standard microbiological diagnosis was performed in all cases for classification of patients. Serum parameters including C-reactive protein (CRP) serum levels and white blood cell (WBC) count were included, and the periprosthetic tissue was immunostained for C9 presence. The amount of C9 tissue staining was evaluated in septic versus aseptic tissue and the amount of C9 staining was correlated with the different pathogens causing the infection. To exclude cross-reactions between C9 immunostaining and other inflammatory joint conditions, we included tissue samples of a separate cohort with rheumatoid arthritis, wear particles and chondrocalcinosis.

**Results:**

The microbiological diagnosis detected PJI in 58 patients; the remaining 40 patients were classified as aseptic. Serum CRP values were significantly increased in the PJI cohort. Serum WBC was not different between septic and aseptic cases. We found a significant increase in C9 immunostaining in the PJI periprosthetic tissue. To test the predictive value of C9 as biomarker for PJI we performed a ROC analyses. According to the Youden’s criteria C9 is a very good biomarker for PJI detection with a sensitivity of 89% and a specificity of 75% and an AUC of 0.84. We did not observe a correlation of C9 staining with the pathogen causing the PJI. However, we observed a cross reactivity with the inflammatory joint disease like rheumatoid arthritis and different metal wear types. In addition, we did not observe a cross reactivity with chondrocalcinosis.

**Conclusion:**

Our study identifies C9 as a potential tissue-biomarker for the identification of PJI using immunohistological staining of tissue biopsies. The use of C9 staining could help to reduce the number of false negative diagnoses of PJI.

## Introduction

The complement pathway is one of the important mechanisms of the innate immune system to fight infections (1). The complement signaling pathway can be activated in three different ways, the classical, the lectin and the alternative signaling pathway (2, 3), all activating the enzymatic C3-convertase (4, 5). The cleavage of C3 starts a protein cascade, which generates the membrane attack complex (MAC) (6, 7). The MAC complex kills bacteria by destroying the bacterial membrane (8–11). In a study from 2018, it was shown that the activation of the terminal complement pathway during periprosthetic joint infections (PJI) can be used as a marker for the detection of PJI. In this study, C9 showed the most promising results (12).

A PJI is a serious complication after implantation of an endoprosthesis. The incidence is about 1% in all implanted hip and 2% in all implanted knee endoprosthesis. During the revision surgery, the probability of a PJI increases, as well as after a previous infection of the respective joint. Some comorbidities also influence the predisposition of the patient to develop a PJI (13). Typical signs of PJI are pain, swelling and warming of the skin around the joint, as well as fever (14–17), increased serum C-reactive protein (CRP) and in some cases serum leucocyte count (WBC) (18).

A wide range of pathogens can cause PJI. The most frequent pathogens found in PJI are from the *Staphylococcus* genus, especially *Staphylococcus aureus* and *Staphylococcus epidermidis* (19–21). *Streptococcus* spp. or *Enterococcus* are also common pathogens in PJI (20–22). Less frequently, gram-negative bacteria such as *Escherichia coli*, *Enterobacter* spp., *Klebsiella* spp. or *Pseudomonas* spp. can be found in PJI (23). The bacteria can occur in a monomicrobial infection or in combinations in a polymicrobial infection (24).

Early identification of PJIs is important to prevent sepsis and severe tissue damage. Currently, the standard of diagnostic is based on the evaluation of clinical symptoms, analysis of radiolucent lines in an X-ray and the microbiological diagnosis of tissue and fluid samples according to the MSIS criteria (25). There is currently no standard biomarker for PJI identification, but serum inflammation markers are considered as indicators. However, especially for the identification of low-grade infections, serum inflammation markers are not reliable (26). Some PJIs are not detected as infections and diagnosed as aseptic implant failure in case of culture negative PJIs (CN-PJI). The current rate of CN-PJI is about 1- 42% (26–31). Therefore, there is a need for biomarkers to facilitate a more secure identification of PJIs. Some biomarkers for more secure identification of PJIs have been proposed such as synovial fluid alpha-defensin (32, 33) or synovial fluid CRP (34, 35) concentration. CRP belongs to the acute-phase proteins and various clinical studies have already investigated the use of synovial fluid CRP levels as a biomarker for the detection of infections (36–44). Póvoa et al. described that the synovial fluid CRP concentration can be significantly increased during infections. However, some non-infected patients showed also increased synovial fluid CRP-levels, suggesting a possible interference of other inflammatory processes (40). Therefore, CRP can serve as a general indicator of inflammation, but cannot clearly distinguish between aseptic and septic inflammation and thereby facilitate identification of infections.

Another proposed biomarker for PJI is the synovial fluid alpha-defensin concentration. Alpha-defensin is an antimicrobial peptide mainly released by neutrophils (45–47). The synovial fluid alpha-defensin immunoassay has been described to identify PJI with a specificity of 95% and sensitivity of 100% (32, 48–50). However, the synovial fluid alpha-defensin concentration can also be elevated in the presence of wear particles or metallosis (12, 32, 51, 52) and in the presence of crystal deposits (53). Furthermore, the high costs of this detection method together with the cross reactivity in case of wear induced implant loosening (54) result in a demand for alternative biomarkers to secure the diagnosis of PJIs.

We have previously shown that C9 could be a potential indicator for PJI in periprosthetic tissue using immunohistochemical staining (12). The main limitation of the study was the low patient number included in this study and the restriction to shoulder implants. Therefore, in this study we aim to validate the immunostaining for C9 in the periprosthetic tissue for PJI identification using a larger cohort and periprosthetic tissue from knee and hip revisions, as well as testing the cross-reactivity with other inflammatory joint diseases.

## Material and methods

### Patients

98 patients undergoing a revision surgery on the total hip (THA) or the knee joint arthroplasty (TKA) were included in the present study. Ethical approval for this study was provided by the Institutional Review Board of the Medical School (No 207/17). Informed consent was obtained by the patients prior to inclusion into the study.

The surgeries were performed in the Department of Orthopaedic Surgery. The demographic data (patient age at the date of surgery, implantation time, sex, implant type, previous surgery) where recorded. The implantation time describes the time from the installation of the prosthesis to the removal of the prosthesis due to aseptic or septic reasons. As previous surgery is the number of previous operations on the affected joint described, excluding the implantation of the first implant in this joint.

All patients in this study were treated according to the in-house algorithm for identifying a PJI. This identification is based on the MSIS criteria (55). Infections were identified if the major criteria applied: at least two positive cultures of the same organism in tissue cultures or the minor criteria were increased. The minor criteria included:

• Medical history was suggestive of infection
• Serum c-reactive protein level was above the threshold of 5 mg/L
• Serum white blood cell count was above the threshold of 10 Gpt/l
• Histology showed typical inflammatory signs e.g. neutrophils

Based on the available microbiological, histopathological, and clinical findings, patients were classified as septic (PJI present) and aseptic (PJI absent). Exclusion criteria were the presence of inflammatory joint diseases, such as rheumatoid arthritis, gout, chondrocalcinosis or severe metallosis.

To test for cross reactivity of C9 antibody staining wit inflammatory joint diseases, we included another control cohort of chondrocalcinosis (N=14) and rheumatoid arthritis (N= 11) patients coming for primary endoprosthesis implantation, and patients with aseptic implant loosening and an extensive amount of wear particles (N=33) The patients with aseptic implant loosening due to wear particles were further separated in patients with CoCr wear (N= 10), with Ti/Pe wear (N=16) and ceramic on ceramic (N=7) The samples for chondrocalcinosis (CC), rheumatoid arthritis (RA) and wear particles were declared as aseptic as no pathogen was identified in the microbiological diagnostic.

### Microbiological diagnostic testing

Periprosthetic tissue samples were minced into pieces and homogenized in an Ultra-Turrax Drive control disperser (IKA^®^-Werke GmbH & Co. KG, Germany) at 6,000 rpm for 2 min in interval direction change. Briefly, the homogenized samples were inoculated on agar plates: Columbia agar with 5% sheep blood (Becton Dickinson, Heidelberg, Germany), chocolate agar and Schaedler agar (Oxoid, Munich, Germany) under aerobic conditions with 5% CO_2_ and anaerobically at 35± 1°C. Additionally, the samples were inoculated in thioglycolate and Schaedler broth (bioMérieux, Marcy L’Etoile, France) at 35 ± 1°C for 14 days. The identification of pathogens was performed by MALDI-TOF MS (VITEK^®^ MS, bioMérieux, Marcy L’Etoile, France).

### Immunohistochemically staining

The periprosthetic tissues were fixed overnight in 4% paraformaldehyde. The tissue was embedded in paraffin and cut into 4-µm sections. The immunofluorescent staining was performed using a C9-antibody (Abcam, Cambridge, England). The corresponding IgG antibody was used as isotype control for the staining. The demasking of the epitopes was performed 0.25 mg/ml pepsin for 45 min at 37°C. The dilution of the antibody was performed in 4% BSA, for the C9-antibody a dilution of 1:500 and for the IgG isotype control a dilution of 1:1000 was used. As secondary antibody an Alexa Fluor^®^555 anti-rabbit (Abcam, Cambridge, England) (1:200 in PBS). The area of red immunofluorescence was calculated as the percentage of the total tissue area in 400x magnification pictures. The average of 3 representative pictures for each sample was calculated using ImageJ (version 1.5; National Institutes of Health, Bethesda, MA, USA). The amount of fluorescence staining was adjusted to the IgG control for the whole cohort and residual staining of the IgG controls for each picture were subtracted to ensure the reliability of evaluated C9-antibody staining.

### Statistical analysis

Statistical analysis was performed using GraphPad Prism (version 8; GraphPad Software, San Diego, CA, USA). Normality and log normality tests were performed to calculate the normal distribution; in the case of a nonparametric distribution, the mean ± SEM using descriptive statistics were calculated. Kruskal-Wallis test and Mann-Whitney test were used to test statistical significance. The ROC curve was performed using the statistical program for ROC curves in Prism. Youden’s criteria were applied to calculate the threshold for the biomarker.

## Results

### Serum CRP and WBC count are indicators for periprosthetic joint infection

We included 98 patients in the PJI versus aseptic cohort in this study. The patients were divided into septic and aseptic according to the described classification based on the MSIS criteria (56). We included 40 aseptic and 58 septic samples. The reasons for aseptic revision were wear induced loosening, malpositioning or luxation of the implant. The periprosthetic tissues of hip arthroplasty (THA) and total knee arthroplasty (TKA) revisions from male and female patients were included. The average age was comparable in both groups (**Table 1**). In the septic group, we included 36 cases of septic THA and 22 septic TKA. In the aseptic group, we included 18 THA and 22 TKA revisions. The implantation time was lower in the septic in comparison to the aseptic group. The number of previous surgeries at the respective joint is indicated in (**Table 1**). Both groups included patients with several previous surgeries at the site of implantation. (**Table 2**) summarizes the comorbidities of the included patients. PJI patients had more often diabetes (21/58), renal insufficiency (11/58), COPD (6/58) and heart insufficiency (6/58) compared to the aseptic cohort.

**Table 1:** Demographic data.

Cohort	Patients	Sex	Age [yr]	Location	Implant. time [m]	No. of previous revisions
Septic	58	♂ 36	72 ± 9	TKA: 22	38 ± 63	1 (21/53); 2 (19/53); 3
		♀ 22		THA: 36		(8/53); 4 (4/53); 7 (1;53)
Aseptic	40	♂ 18	71 ± 10	TKA: 22	95 ± 76	1 (18/35); 2 (10/35); 3
		♀ 22		THA: 18		(4/35); 4 (2/35); 10 (1/35)

**Table 2:** Comorbidities.

	Septic (N=58)	Aseptic (N=40)
**Diabetes**	**21**	**17**
Osteoporosis	2	1
**Renal insufficiency**	**11**	**6**
**COPD**	**6**	**1**
**Heart insufficiency**	**6**	**1**
Asthma	1	4
Other	11	5

The bold values belong to the relevant comorbidities.

**Figure 1:**
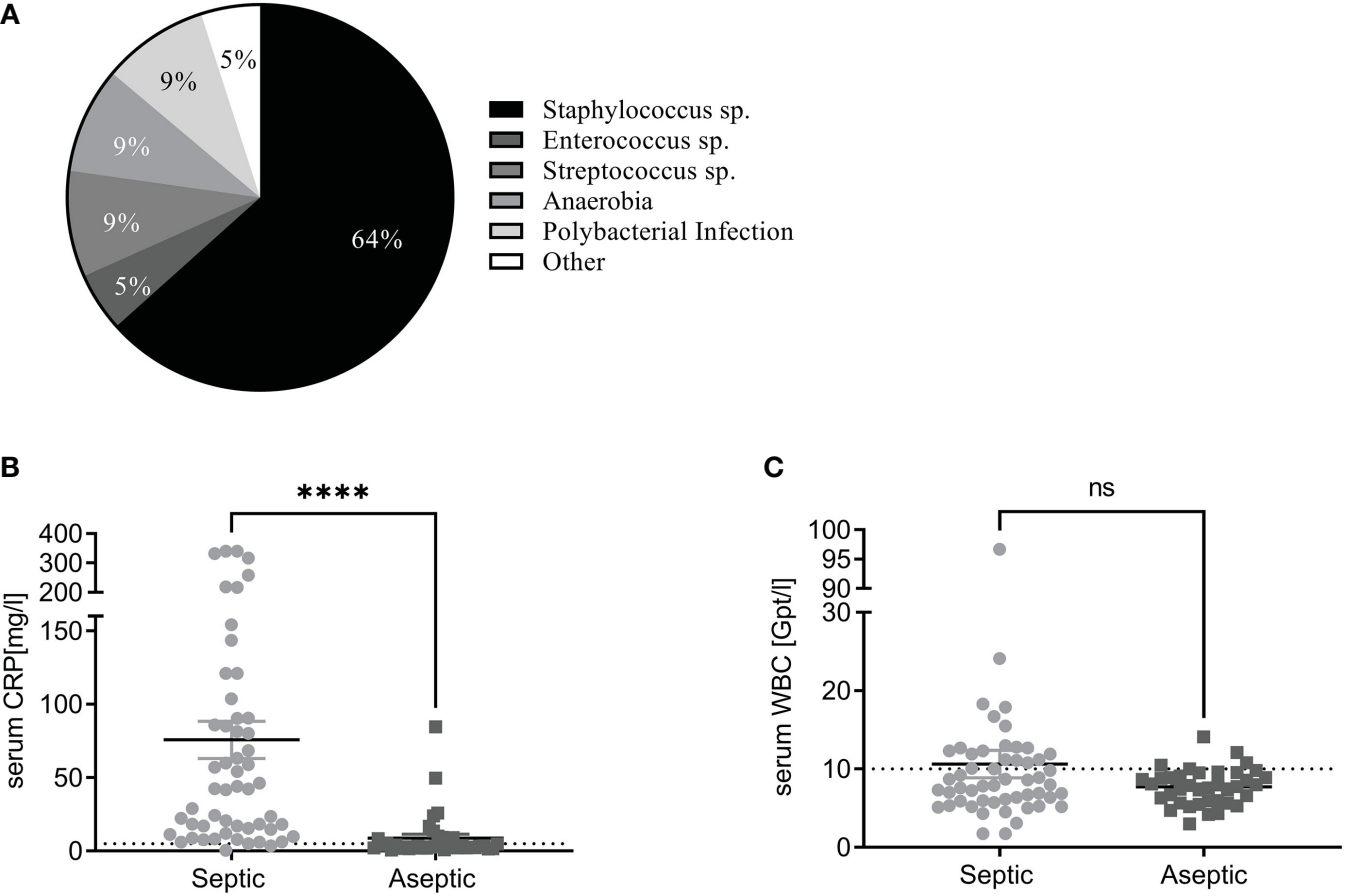
Serum CRP and WBC count are indicators for periprosthetic joint infection **(A)** Pie chart of the identified pathogen spectrum. *Staphylococcus* spp. was the major pathogen that was identified by the microbiological diagnostic. Other bacteria were less frequently detected. **(B)** Serum C-reactive protein (CRP) values (mg/l) in septic (light grey) and aseptic (dark grey) groups. The serum CRP value of the septic cohort was significantly increased in the septic compared with the aseptic cohort (Mann-Whitney test: p<0.0001). The pathologic threshold is 5mg/L is indicated as a black dashed line. **(C)** The mean value of the serum white blood cell (WBC) count (Gpt/l) was not different between both cohorts (Mann-Whitney test: p= 0.237). Most patients exhibited a WBC count below the pathological threshold of 10 Gpt/l (black dashed line) for all tested groups. ****P < 0.0001, ns, not significant.

The bacterial spectrum identified by the microbiological diagnostic showed that *Staphylococcus* spp. infections occurred most frequently (37/58), followed by *Streptococcus* spp. (5/58), anaerobic bacteria (5/58), polymicrobial infections (5/58) and *Enterococcus* spp. (3/58) (**Figure 1A**). The CRP serum level was significantly increased in the septic group compared to the aseptic cohort (**Figure 1B**) (p < 0.0001). However, not all patients within the septic (42.40 ± 12.66 mg/l) cohort showed a pathological CRP value of more than 5 mg/l. Importantly, 12 out of 40 aseptic (3.3 ± 2.61 mg/l) patients also exhibited an increased CRP value. The serum WBC count was not significantly (p = 0.2373) changed between the aseptic (7.84 ± 0.37 Gpt/l) and the septic (8 ± 1.76 Gpt/l) (**Figure 1C**).

### C9 immunostaining of periprosthetic tissue could serve as potential biomarker for PJI identification

**Figure 2:**
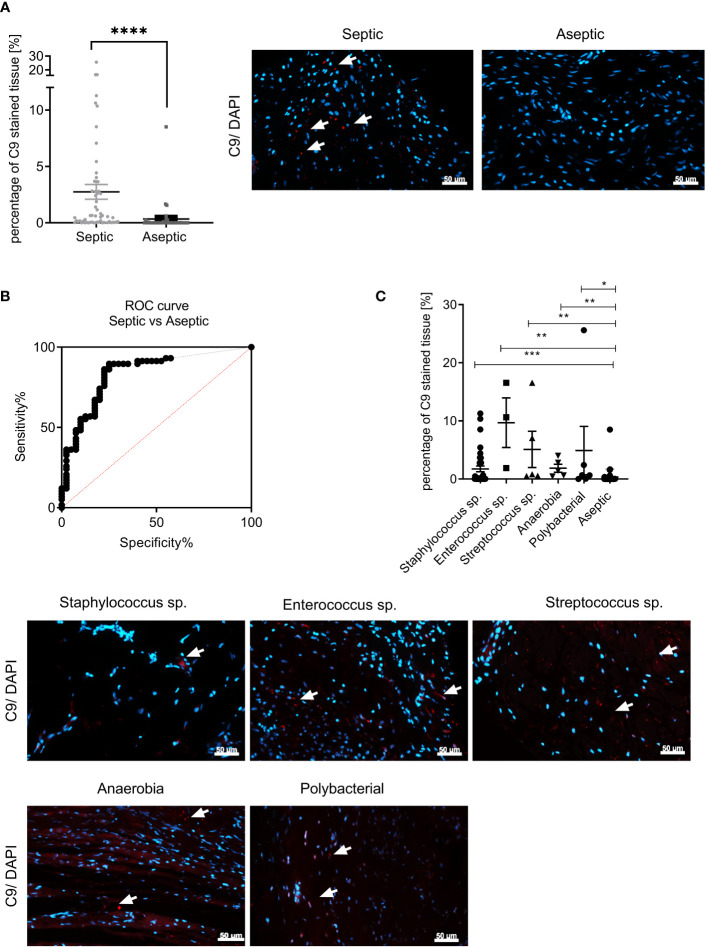
C9 immunostaining of periprosthetic tissue could serve as potential biomarker for PJI identification. **(A)** The statistical analysis of the percentage of red fluorescence within the total tissue area (C9 immunostaining) of the aseptic (dark grey, N = 40; n = 120) and septic (light grey, N = 58, n = 174) cohort can be seen as a scatter dot plot. The values indicate the percentage of C9 immunostaining. Three images for each patient sample were analyzed (Mann-Whitney test: p < 0.0001). A representative picture for a septic (left) and an aseptic (right) sample are depicted. (nuclei: blue, anti-C9 immunostaining: red). White arrows indicate C9 stained tissue area. The scale bar (white bars) indicates 50 µm and pictures were taken at 400x magnification. **(B)** The ROC-curve for individual percentages of C9 stained tissue area for septic and aseptic samples. The area under the curve is 0.84. The sensitivity of 89% (95% of Cl: 78.83% to 96.11%) and the specificity of 75% (95% of Cl: 58.80% to 87.31%) were calculated using the Youden’s criteria (red dashed line). **(C)** Representative immunohistochemical staining of C9-antibody (red) in periprosthetic tissue of patients with an PJI caused by *staphylococcus* sp., *streptococcus* sp., *enterococcus* sp., anaerobia and polymicrobial infections compared to the aseptic cohort. The values indicate the percentage of C9 immunostaining. Three images for each patient sample were analyzed (*staphylococcus*: N = 37; n = 111, *enterococcus* N = 3; n = 9; *streptococcu*s: N = 5; n = 15; *anaerobia*: N = 5; n = 15; polymicrobial infection: N = 6; n = 18; aseptic: N = 58, n = 174). White arrows indicate C9 stained tissue area. The scale bar (white bars) indicates 50 µm and pictures were taken at 400x magnification. No pathogen dependent influence on the percentage of C9 immunostained tissue was observed (Kruskal-Wallis test with Dunn’s *post hoc* test: p<0.0001). *p < 0.05, **p < 0.01, ***p < 0.001, **** p < 0.0001; n, total number of measurements; N, number of samples, ns, not significant.

C9 immunostaining has been proposed to be a reliable marker for the identification of PJI using tissue biopsies (12). To validate this observation we stained the periprosthetic tissue of hip and knee endoprosthesis revision surgeries due to PJI or aseptic implant loosening or malpositioning for C9. We observed significantly more C9 immunostaining in the septic tissue (mean ± SEM: 2.74% ± 0.65%) (**Figure 2A**) compared to the aseptic cohort (mean ± SEM: 0.34% ± 0.22%) (p<0.0001).

To investigate the predictive value of C9 staining, a receiver operating curve (ROC) curve analysis using the respective C9 immunostained area for each sample, was performed (**Figure 2B**). Here, we plotted the percentage of C9 positive area in the septic tissues (sensitivity) against the percentage of C9 positive area in the aseptic tissue (100%-specificity). The area under the curve (AUC) of the ROC analysis is 0.84. Using the Youden’s criteria the threshold for the sensitivity and the specificity (red dashed line) was calculated. The sensitivity for the C9 staining was at 89% (95% of Cl: 78.83% to 96.11%), while the specificity was at 75% (95% of Cl: 58.80% to 87.31%).

To investigate whether there is a pathogen-dependent increase of C9 tissue staining we divided the PJI samples according to the identified pathogen into Staphylococcus spp. (N= 37, 1.75% ± 0.49%), *Enterococcus* spp. (N= 6, 9.69% ± 4.27%), *Streptococcus* spp. (N= 3, 5.1% ± 3.13%), Anaerobia (N= 5, 1.86% ± 0.68%) and Polymicrobial infections (N= 5, 4.9% ± 4.15%) and depicted the C9 stained tissue area for each sample. The statistical analysis of C9 immunostaining showed no difference in the amount of C9 tissue staining with regard to the different pathogens detected by the microbiological diagnostic. (**Supplementary Figure 1D**). Next we co compare the amount of C9 immunofluorescent staining with the aseptic cohort. We observed a significant difference for all pathogens compared to the aseptic tissue, indicating that C9 could be a universal biomarker (**Figure 2C**).

### Anti-C9 immunostaining showed no cross reactivity with chondrocalcinosis and most wear particle types

Since other proposed biomarkers for PJI, such as alpha-defensin, showed cross-reactions with tissue residues of hemorrhaging or abrasive wear, we investigated the cross reactivity of C9 with different inflammatory joint conditions. We included periprosthetic tissue samples from patients with chondrocalcinosis (CC) (14 samples), rheumatoid arthritis (RA) (11 samples) and different types of wear particles (33 samples) (**Table 3**). The wear particles resulted from the bearing coupling and therefore, the samples were divided into patients with cobalt-chromium (CoCr) (11 samples), titanium-plastic (Ti+Pe) (15 samples) and ceramic (CoC) wear (7 samples). The average age the CC cohort was 65 ± 10, RA had an average age of 61 ± 14 and the wear particle cohort had an average age of 65 ± 11. In the CC cohort 5 samples were from male patients while 9 samples were from females. All periprosthetic tissue sections were collected from the knee. Three samples were from male patients in the RA cohort, while 7 samples were from female patients. In the cohort of the wear particle cohort 17 samples were used from male patients, while 16 samples were used from female patients. The periprosthetic tissue was collected from the hip and the knee (**Table 3**).

**Table 3:** Patient characteristics of septic, CC, RA and wear particles cohort.

Cohort	Number	Sex	Age [yr]	Location	CRP [mg/l]	WBC [Gpt/l]
Septic	58	♂ 36 ♀ 22	72 ± 9	TKA: 22 THA: 36	38 ± 63	1 (21/53); 2 (19/53); 3
CC	19	♂ 15 ♀ 4	67 ± 11	TKA: 19 THA: 0	(8/53); 4 (4/53); 7 (1;53)	(8/53); 4 (4/53); 7 (1;53)
RA	17	♂ 2 ♀ 15	66 ± 18	TTK: 22 TsA: 36	95 ± 76	1 (18/35); 2 (10/35); 3
				TTK: 2 TSA: 2		
Wear particles	16	♂ 5 ♀ 11	70 ± 13	TKA:4 THA:12	13 ± 34.93	7 ± 1,34

**Figure 3:**
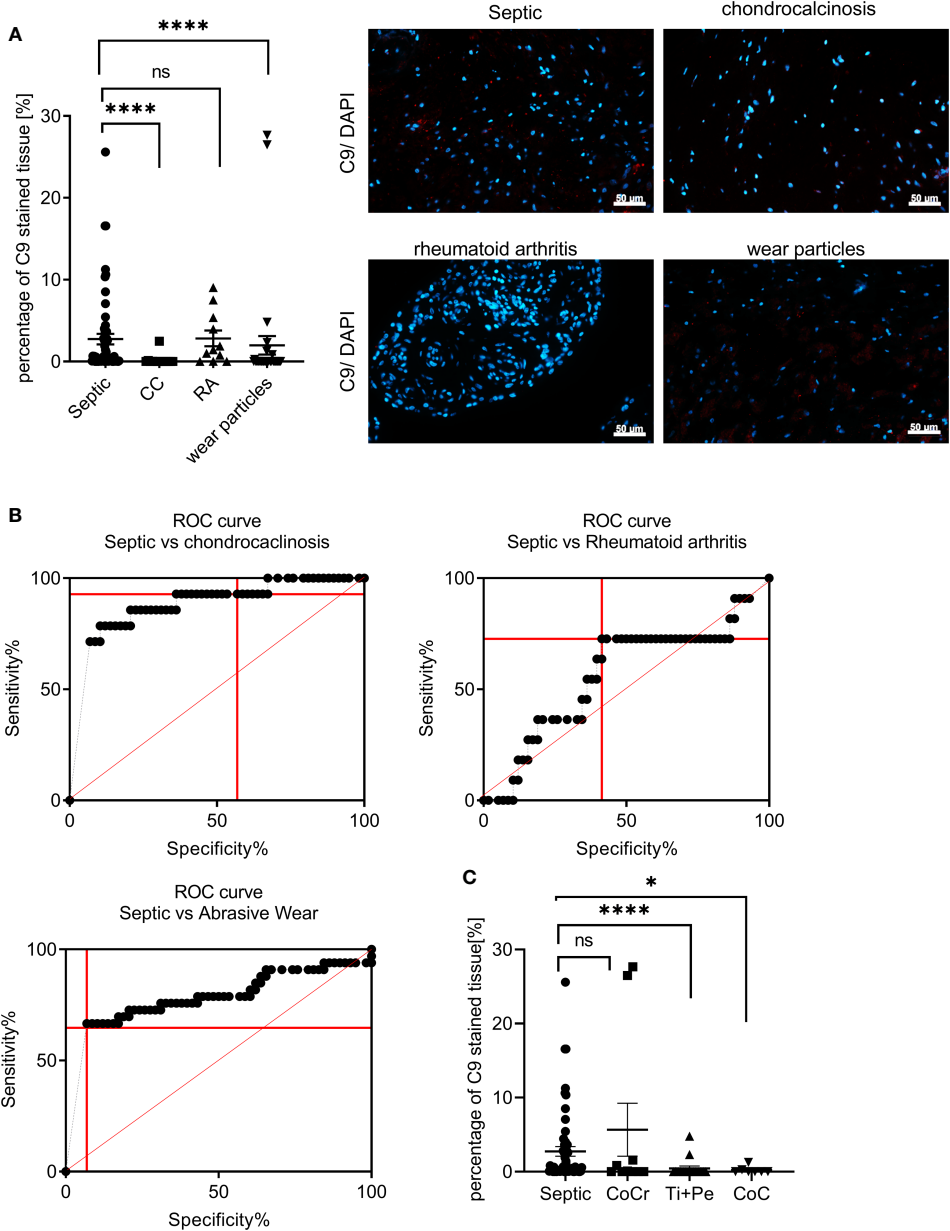
Anti-C9 immunostaining showed no cross reactivity with chondrocalcinosis and most wear particle types **(A)** Representative immunohistochemical stainings of periprosthetic tissue of patients with PJI (septic), chondrocalcinosis, rheumatoid arthritis and wear particles with the C9-antibody (red) are shown. The values indicate the percentage of C9 stained tissue area. Three images for each patient sample were analyzed (septic: N = 58; chondrocalcinosis: N = 14; rheumatoid arthritis: N = 11; wear particles: N = 33). White arrows indicate C9 stained tissue area. The scale bar (white bar) indicates 50 µm and pictures were taken at 400x magnification. (ANOVA: *post hoc*, septic vs CC (p < 0.0001), septic vs wear particles (p < 0.0001), septic vs RA (p > 0.999). **(B)** The ROC-curve for individual percentages of C9 stained tissue area for septic and aseptic samples. Using the Youden’s criteria the best ratio of sensitivity to specificity was selected for each condition (red dashed line). The ROC-curve for septic versus CC group showed an area under the curve of 0.88. The sensitivity was 92.86% (95% of CI: 66.13% to 99.82%) while the specificity was 56.90% (95% of Cl: 43.23% to 69.84%).The ROC-curve for septic versus RA group showed an area under the curve of 0.56. Using the Youden’s criteria the best ratio of sensitivity to specificity was selected (red dashed line). The sensitivity was 72.73% (95% of Cl: 39.03% to 93.98%while the specificity was 58.62% (95% of Cl: 44.93% to 71.40%). The ROC-curve for septic versus wear particles had an AUC of 0.77. Using the Youden’s criteria the best ratio of sensitivity to specificity was selected (red dashed line). The sensitivity was 64.71% (95% of Cl: 46.49% to 80.25%while the specificity was 93.1% (95% of Cl: 83,27% to 98,09%). **(C)** Representative immunohistochemical staining of the C9-antibody (red) in periprosthetic tissue of patients with PJI (septic) or various wear types can be seen. The values indicate the percentage of C9 stained tissue (septic: N = 58; CoCr: N = 10; Ti+PE: N = 16; CoC: N = 7). Three images for each patient sample were analyzed. White arrows indicate C9 stained tissue area. The scale bar (white bars) indicates 50 µm and pictures were taken at 400 x magnification. (ANOVA: *post hoc*). *p < 0.05, ****p < 0.0001;.

We stained the periprosthetic tissue from the different cohorts for C9 and depicted the amount of C9 stained tissue area in the diagram. Patients with CC showed a C9 tissue staining of mean ± SEM: 0.2% ± 0.18%, RA mean ± SEM: 2.83% ± 0.96%, and wear particles had a mean ± SEM: 1.98% ± 1.1% (**Figure 3A**). The amount of C9 immunostaining was significantly higher in the infected periprosthetic tissue compared to CC synovial tissue (p < 0.0001). C9 staining was also significantly increased in case of wear particles in the periprosthetic tissue compared to septic (p <0.0001). However, no significant difference in the amount of C9 staining in septic tissue compared to RA synovial tissue was observed (p > 0.9999).

To test the predictive value of C9 as biomarker in case of the above-mentioned inflammatory joint diseases, we performed a ROC analyses for C9 tissue staining for each condition. The discrimination between CC and septic using C9 staining showed a good predictive value (AUC: 0.88) (**Figure 3B**). The separation of the amount in C9 staining between septic and RA was lower and showed a lower predictive value. The separation between septic and wear particle containing tissue using C9 staining, however, exhibited an AUC: 0.78.

Since we observed marked differences in the percentage of C9 stained tissue area in the presence of wear particles, we investigated whether the abrasion type had an influence on the amount of C9 stained tissue area (**Figure 3C**). When comparing the C9 stained area in the septic samples with Ti+ PE (mean ± SEM: 0.45% ± 0.32%, p < 0.0001), and Ceramic on Ceramic (CoC) (mean ± SEM: 0.22% ± 0.18%, p = 0.0156) a clear separation was observed. In the presence of CoCr (mean ± SEM: 5.66% ± 3.57%, p = 0.4095), however, no statistical significance was determined, indicating a cross-reaction between the C9 antibody staining with CoCr wear particles in tissues (**Figure 3C**).

## Discussion

The current study investigated the use of C9 immunostaining of periprosthetic tissues as a biomarker for PJI using patients undergoing THA or TKA revision due to septic or aseptic implant revision. As expected, patients with more than two previous surgeries at the respective joint exhibited more frequently septic (**Table 1**) (57–59). Comorbidities such as congestive heart failure, diabetes, depression, anemia, chronic lung disease, obesity, rheumatologic disease, kidney disease, and pulmonary circulatory disorders were established as typical risk factors for developing a PJI (60–62). These observations were corroborated in our septic patient cohort (**Table 2**). The patients in the septic cohort exhibited more often comorbidities compared to the patients in the aseptic cohort. Especially diabetes, renal insufficiency, heart failure, and COPD were more common in the septic group than in the aseptic group.

PJI has been associated with an increase in systemic inflammatory serum markers (14). We observed an increased serum CRP level in most patients in the septic cohort (**Figure 1B**). However, the discrimination was not clear, as also some aseptic patients exhibited an increased CRP value, as well as not all septic patients exhibited a pathological serum CRP level. Other studies already showed that the predictive value of the serum CRP level for identification of PJI is low (63, 64). The serum WBC count did not show a significant difference between the septic and aseptic cohort in our study. This observation has already been described in another study (65).

In our cohort, we mostly identified pathogens from the *Staphylococcus* genus in the septic samples (64%) (**Figure 1A**). This is consistent with other studies, showing that *S. aureus* and *S. epidermidis* occur most frequently in PJIs (19, 21, 66). PJIs caused by *Streptococcus* spp. and *Enterococcus*, as well as polymicrobial infection and anaerobic pathogens were detected with lower frequency. This is also corroborated by others, as Enterococcus spp. and Streptococcus spp. occur less common in PJIs (66) as well as anaerobia (67, 68).

The MSIS criteria (25) describe that one major factor for the identification of a PJI are two tissue cultures positive for the same pathogen. However, contamination during sampling, the presence of a biofilm on the implant or pre-treatment with antibiotics affects the detection of pathogens using the routine microbiological diagnostic methods (69–72). These influencing factors increase the risk of culture negative (CN)-PJI, leading to an inadequate treatment of the patient (26, 27). Therefore, minor factors such as serum CRP, synovial fluid WBC or histopathology of tissue samples are used for the verification of the diagnosis. Our data indicate that the serum CRP and the serum WBC count in our cohort was of low predictive value for septic identification. The serum CRP, as well as the serum WBC count, can be increased during other acute inflammatory disease, making both parameters unreliable indicators for PJI (65, 73, 74). The prevalence of CN-PJI is about 1- 42% (28–31) raising the need for a biomarker to decrease the number of CN-PJIs.

The complement pathway is an important mechanisms of the innate immune system to fight infections (1). It was shown that previously that C3, C5 and C9 were detectable by immunostaining in the periprosthetic tissue in a cohort of shoulder PJI patients (12). Here, C9 immunostaining of the tissue showed the highest sensitivity and specificity to discriminate between the septic and the aseptic cohort. Therefore, the authors suggested that C9 immunostaining could be used as a possible biomarker for the identification of PJI (12). We observed again a significant increase in C9 immunostaining of the periprosthetic tissue of PJI patients, compared to aseptic loosening (**Figure 2A**). The ROC curve for percentage C9 immunostaining in the tissue comparing aseptic tissues with septic tissues resulted in an AUC value of 0.84. The Youden’s index indicated a sensitivity of 89.66% and specificity of 75% for C9 as biomarker for PJI. Therefore, according to the classification of biomarkers (75), C9 immunostaining of periprosthetic tissue could be an excellent biomarker for identification of PJI. However, the synovial fluid alpha-defensin ELISA has been described to exhibit a sensitivity of 100% and specificity of 95% for identifying PJI (32, 48–50). It is suggested, that synovial fluid alpha-defensin detection should be used as a supplementary diagnostic method in cases in which PJI cannot be diagnosed clearly, that are nevertheless suspected to be septic (54).

C9 immunostaining exhibited a sensitivity of 89.6%, indicating that not all septic tissues showed C9 immunostaining. A reason might be that the complement pathway is mainly activated in the early phase of infection (76) and that C9 is not present in later stages of infection. However, we did not find a significant correlation when comparing the implantation time with the percentage of C9 stained periprosthetic tissue (**Supplementary Figure 1B**, p = 0.9985)). In addition, we did not observe a statistical significance for the percentage of C9 stained tissue within the septic cohort, when we divided the PJI cases into early (<3 months after implantation), delayed (3 – 12 months after implantation) and late (>12 months after implantation) infections (**Supplementary Figure 1**). Therefore, we think that the phase of infection is not the reason for some septic samples being negative for C9 immunostaining.

Next, we tested if the percentage of C9 immunostaining might be dependent on the pathogen causing the PJI (**Figure 2C**). Here, we divided the septic cohort based on the bacteria causing the PJI into *Staphylococcus* spp. (66), *Streptococcus* spp., *Enterococcus* spp. (66). and *Anaerobia* (67, 68) as well as polymicrobial PJI (15). No significant difference in the percentage of C9 immunostaining of the periprosthetic tissue was observed between the tested pathogens, indicating that the amount of C9 immunostaining is not pathogen dependent. This finding indicates, that C9 as a potential biomarker can be used independently from factors such as stage of infection, implantation time and pathogen causing the PJI.

Besides not all septic samples being positive for C9 immunostaining, also some aseptic samples were positive for C9 staining, explaining the specificity of 75% for C9 as a biomarker.

As synovial fluid alpha-defensin showed a cross-reactivity in the presence of crystal deposits (53, 77) and abrasive wear (52), we wanted to investigate if the C9 immunostaining in the tissue showed similar cross-reactivity with other inflammatory joint conditions such as chondrocalcinosis (CC), rheumatoid arthritis (RA) and wear particles. We observed no cross-reactivity of C9 immunostaining in the tissue CC and most types of wear particles, while C9 was not able to distinguish between RA and septic. Thurman et al. proposed that some autoantibodies, such as they are present in rheumatoid arthritis (RA), could also activate the terminal complement pathway (78–80). This fact could explain why C9 was also detectable in tissue of RA patients.

Metal particles are known to induce an inflammatory tissue responses (81), therefore it was investigated whether the different wear particle types could also influence the detection of C9 in the periprosthetic tissue (**Figure 3C**). We found that in the presence of titanium and PE particles (Ti+Pe) and ceramic particles (CoC) C9 was mainly present in the septic cohort, but not in the tissue of the respective bearing couplings. In contrast, periprosthetic tissue from patients with CoCr implants showed a similar C9 immunostaining compared to the septic cohort. A study showed that an immune response was elicited in the presence of cobalt, chromium, and nickel, while no increase was detected with titanium particles (82). CoCr particles were shown to exhibit a sever inflammatory response in a mouse model (81), indicating a difference in the inflammatory capacity of different wear partciles and making CoCr particles more inflammatory than others. This observation could explain why only CoCr wear particles showed a cross-reactivity with C9 immunostaining.

As the synovial fluid alpha-defensin immunoassay is not suggested as stand-alone technique for the routine diagnostic, but should rather be applied in cases where the diagnosis of PJI was unclear (54), we propose that C9 immunostaining should be applied to help the decision making in unclear cases of PJI.

One limitation of our study is that the location of the collected periprosthetic tissue was not clearly defined and standardized throughout the cohort. Due to the unclear sampling area, some samples could be closer to the infection site than other samples, explaining the variety in the percentage of C9 immunostaining in the tissue samples. As in this septic cohort more gram-positive bacteria were causing the PJI, it would be interesting include more samples of other pathogens to clearly show whether there might be a pathogen dependent difference in the C9 detection. Furthermore, we did not analyze specifically if low-grade infections could be identified more clearly with the help of C9 staining, to help identify PJI in unclear cases. Further experiments will be needed to validate the possible use of C9 immunostaining for a more reliably detection of CN-PJIs and low-grade infections.

## Conclusion

In this study, we shown that C9 immunostaining is a very good tissue-biomarker for the identification of PJI with biopsy specimens. Due to the high AUC value as well as the high sensitivity and specificity of C9 immunostaining, we propose the use of C9 immunostaining in cases of unclear diagnosis of PJI to secure the treatment suggestion.

## Data availability statement

The raw data supporting the conclusions of this article will be made available by the authors, without undue reservation.

## Ethics statement

The studies involving human participants were reviewed and approved by Institutional Review Board of the Medical School Magdeburg (No 207/17). The patients/participants provided their written informed consent to participate in this study.

## Author contributions

A-KM performed the histological stainings and evaluation of data. JF performed the microbiological diagnostic. SI identified the relevant patients and provided the patient history as well as performed the sampling. PM developed the C9 antibody and discussed data and helped interpreting the results. CL helped writing the manuscript and discussing and interpreting the data. JB made the conceptualization and supervision for the project and wrote the manuscript. All authors contributed to the article and approved the submitted version.

## Supplementary material

Supplementary Figure 1 No correlation between the percentages of C9 stained tissue area with the serum CRP level, implantation time, and stage of infection **(A)** In order to test a potential correlation between the percentage of C9 tissue staining and the serum CRP value of the respective patient. No correlation was observed for the septic and aseptic cohort (Linear regression septic r^2^ = 0.01; 95% Cl = -0.01 to 0.02, p = 0.41; N = 54; aseptic r^2^ = 0.004; 95% Cl = -0.02 to 0.04, p = 0.07; N = 37). **(B)** A potential correlation between the percentage of C9 tissue staining and the implantation time in month the septic and aseptic cohort were compared. No correlation was found for the septic and aseptic cohort (Linear regression: septic r^2^ = 7.08e-008, 95% Cl = -0.02 to 0.02, p = 0.09; N = 53, aseptic septic r^2^ = 0.04, 95% Cl = -0.01 to 0.003, p = 0.26; N = 37). **(C)** Potential influence of infection type (early: <3 months after implantation;, delayed: 3 – 12 months after implantation, months after implantation) on the percentage of C9 tissue staining (early: N = 12, delayed: N = 14, late: N = 27, One-Way-ANOVA with Tukey *post hoc* test F (2, 50) = 1.26, p=0.29). **(D)** Representative immunohistochemical staining of C9-antibody (red) in periprosthetic tissue of patients with an PJI caused by staphylococcus sp., streptococcus sp., enterococcus sp., anaerobia and polymicrobial infections. The values indicate the percentage of C9 immunostaining. Three images for each patient sample were analyzed (staphylococcus: N = 37; n = 111, enterococcus N = 3; n = 9; streptococcus: N = 5; n = 15; anaerobia: N = 5; n = 15; polymicrobial infection: N = 6; n = 18). White arrows indicate C9 stained tissue area. The scale bar (white bars) indicates 50 µm and pictures were taken at 400x magnification. No pathogen dependent influence on the percentage of C9 immunostained tissue was observed (One-way ANOVA with Tukey *post hoc* test: F (4, 51) = 2.682, p=0.0417, which resulted in no significant difference after multiple comparisons between the different groups).
