# Supplementary figures and images for: C9 immunostaining as a tissue biomarker for periprosthetic joint infection diagnosis
Source: Front Immunol. 2023 Feb 21;14:1112188. doi: 10.3389/fimmu.2023.1112188 (PMC9989178; doi:10.3389/fimmu.2023.1112188)

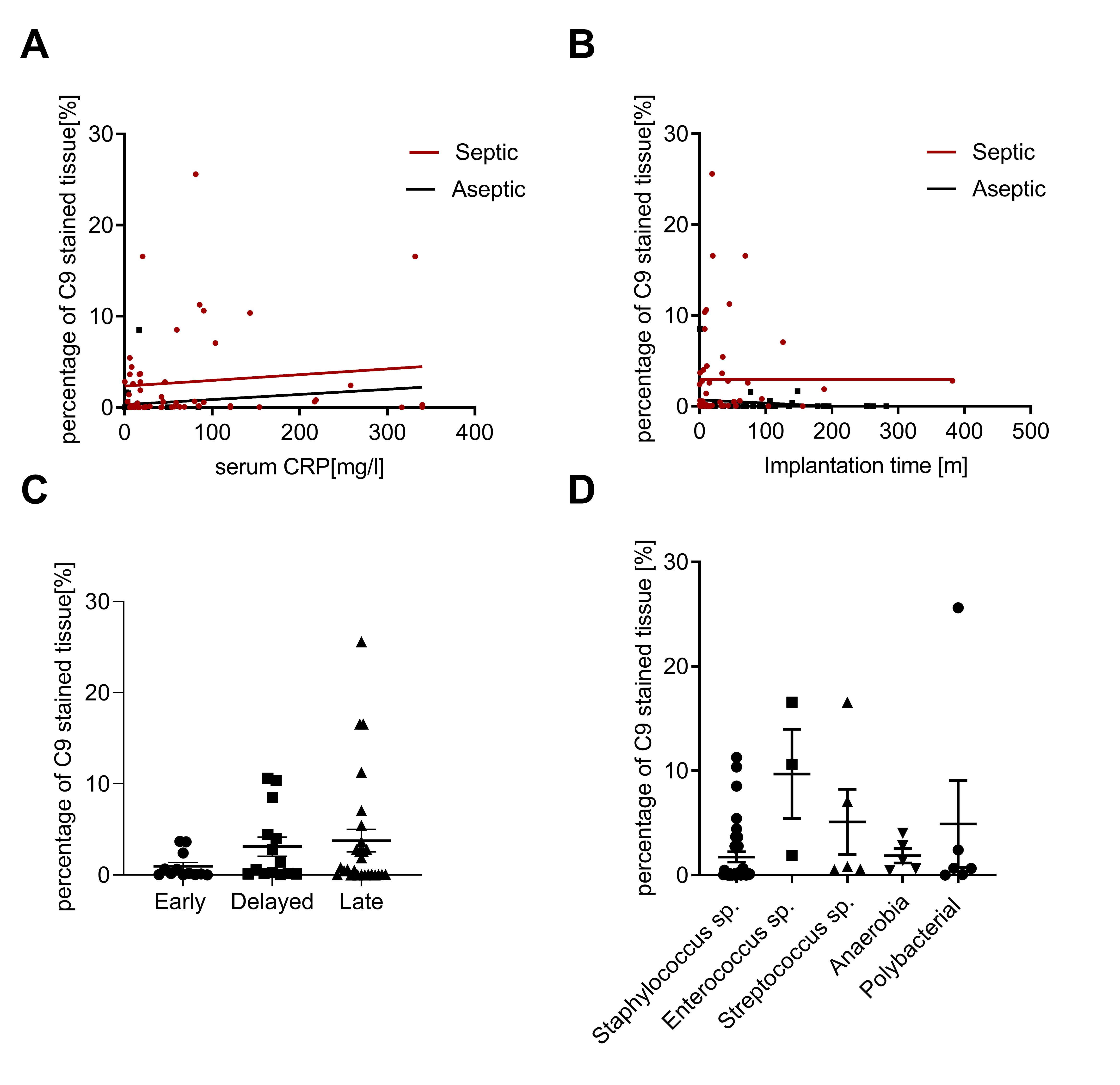

Supplement: Supplementary Figure 1 — No correlation between the percentages of C9 stained tissue area with the serum CRP level, implantation time, and stage of infection (A) In order to test a potential correlation between the percentage of C9 tissue staining and the serum CRP value of the respective patient. No correlation was observed for the septic and aseptic cohort (Linear regression septic r2 = 0.01; 95% Cl = -0.01 to 0.02, p = 0.41; N = 54; aseptic r2 = 0.004; 95% Cl = -0.02 to 0.04, p = 0.07; N = 37). (B) A potential correlation between the percentage of C9 tissue staining and the implantation time in month the septic and aseptic cohort were compared. No correlation was found for the septic and aseptic cohort (Linear regression: septic r2 = 7.08e-008, 95% Cl = -0.02 to 0.02, p = 0.09; N = 53, aseptic septic r2 = 0.04, 95% Cl = -0.01 to 0.003, p = 0.26; N = 37). (C) Potential influence of infection type (early: <3 months after implantation;, delayed: 3 – 12 months after implantation, months after implantation) on the percentage of C9 tissue staining (early: N = 12, delayed: N = 14, late: N = 27, One-Way-ANOVA with Tukey post hoc test F (2, 50) = 1.26, p=0.29). (D) Representative immunohistochemical staining of C9-antibody (red) in periprosthetic tissue of patients with an PJI caused by staphylococcus sp., streptococcus sp., enterococcus sp., anaerobia and polymicrobial infections. The values indicate the percentage of C9 immunostaining. Three images for each patient sample were analyzed (staphylococcus: N = 37; n = 111, enterococcus N = 3; n = 9; streptococcus: N = 5; n = 15; anaerobia: N = 5; n = 15; polymicrobial infection: N = 6; n = 18). White arrows indicate C9 stained tissue area. The scale bar (white bars) indicates 50 µm and pictures were taken at 400x magnification. No pathogen dependent influence on the percentage of C9 immunostained tissue was observed (One-way ANOVA with Tukey post hoc test: F (4, 51) = 2.682, p=0.0417, which resulted in no significant difference after multiple compa [file Image_1.tif]
